# Supplementary material for: Evaluating Ultrasonicator Performance for Cyanobacteria Management at Freshwater Sources
Source: Toxins (Basel). 2023 Mar 1;15(3):186. doi: 10.3390/toxins15030186 (PMC10051689; doi:10.3390/toxins15030186)
Supplement: Supplementary file 1 [file toxins-15-00186-s001.zip › toxins-2088268-supplementary.pdf]

Supplementary Information

Evaluating Ultrasonicator Performance for Cyanobacteria Management at Freshwater Sources

Liam Vaughan, Dean Barnett, Elisa Bourke, Hamish Burrows, Fiona Robertson, Brad Smith, Jenna Cashmore, Michael Welk, Michael Burch and Arash Zamyadi

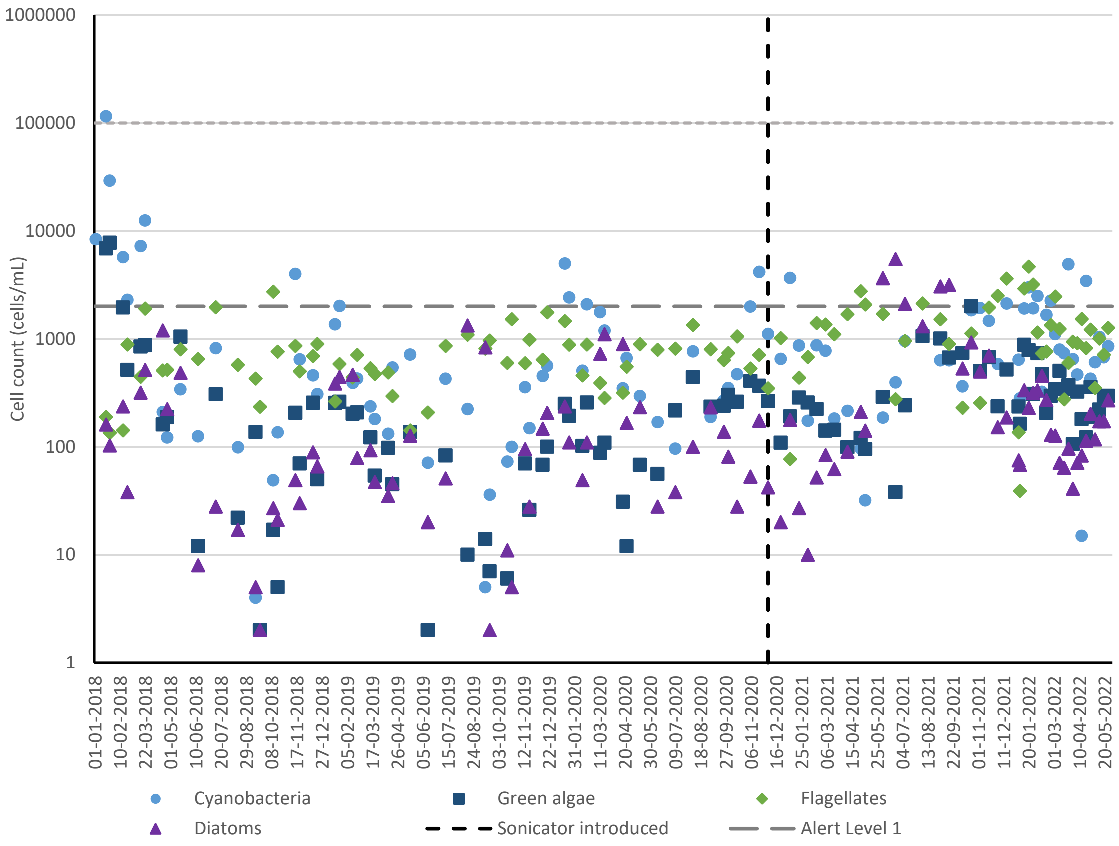

Figure S1. Reservoir C all algal groups timeseries using the reservoir water dataset.

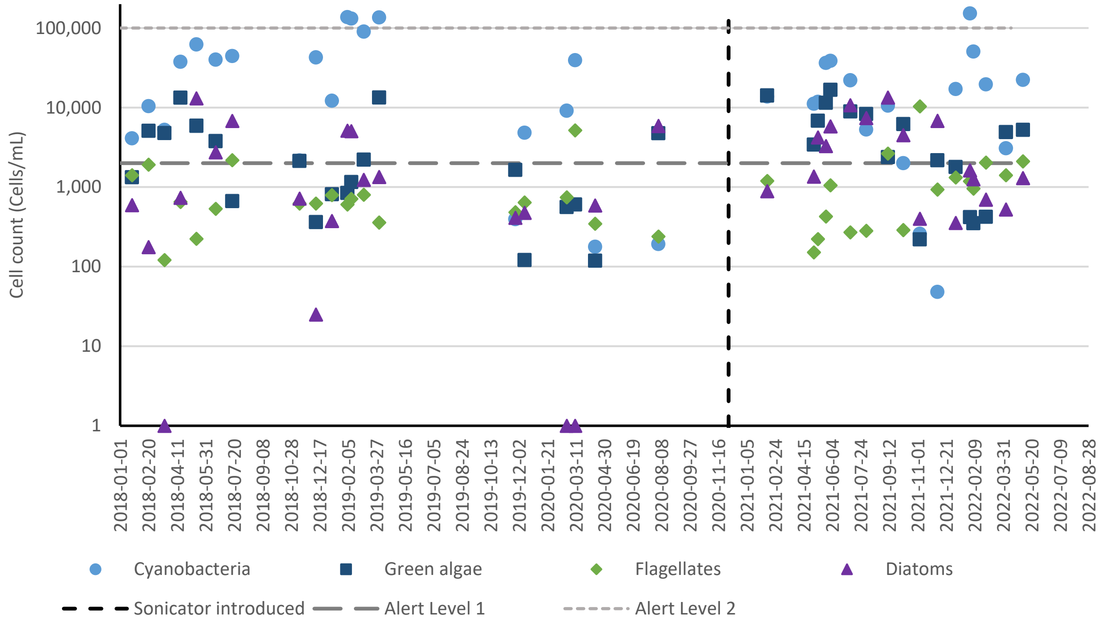

Figure S2. Reservoir B all algal groups timeseries.

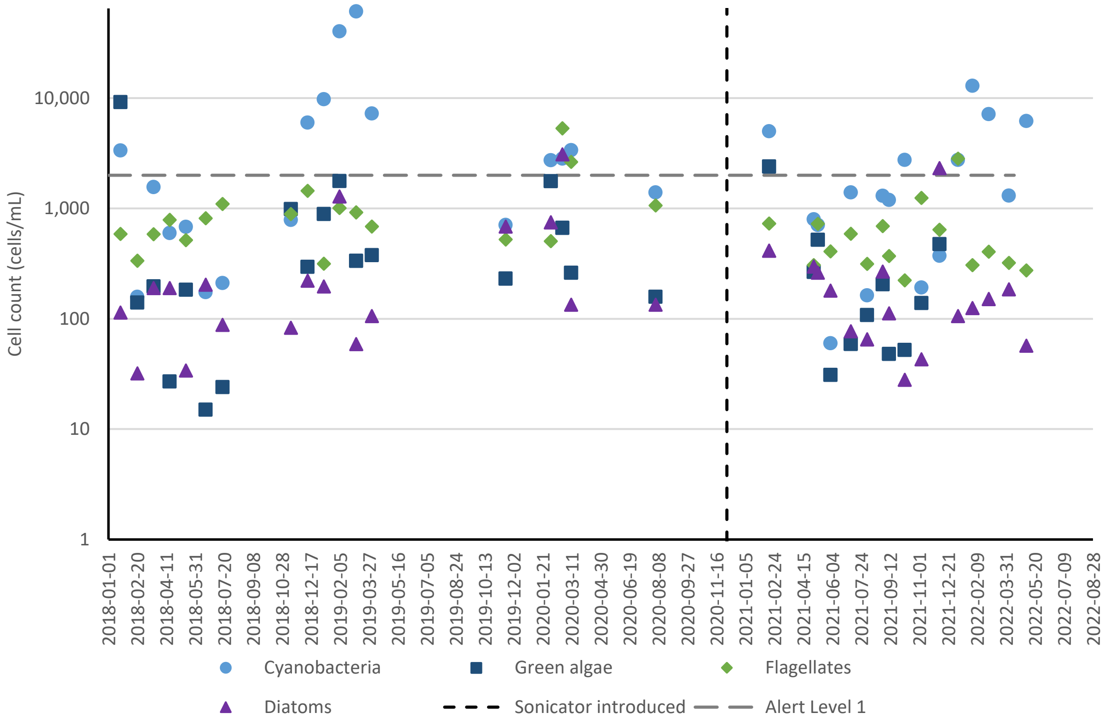

Figure S3. Reservoir A all algal groups timeseries.

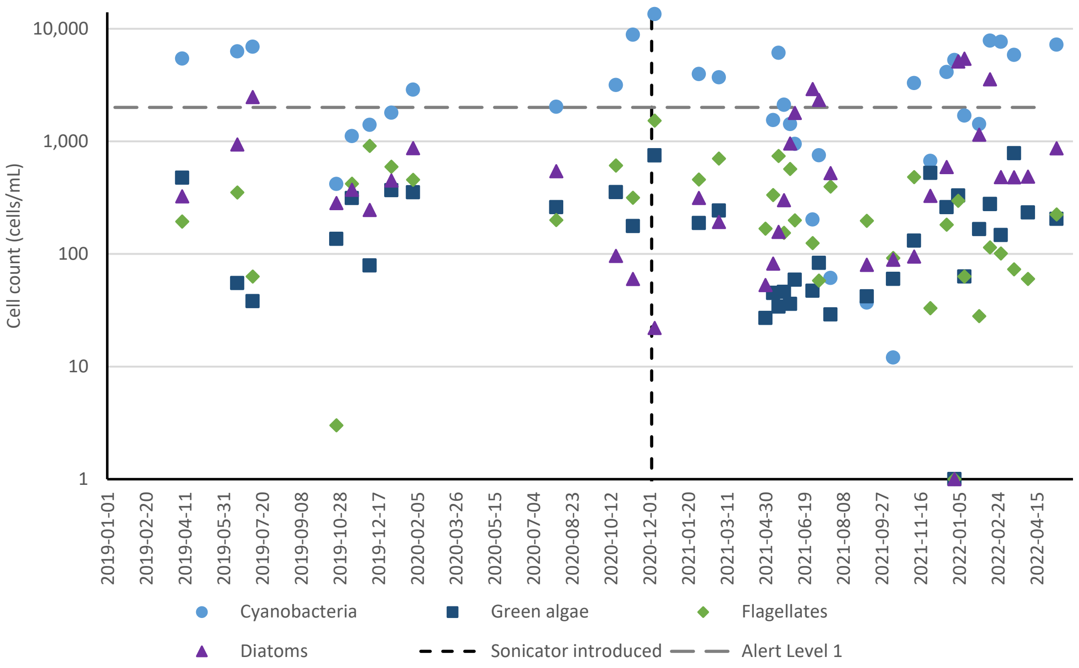

Figure S4. Reservoir D all algal groups timeseries.

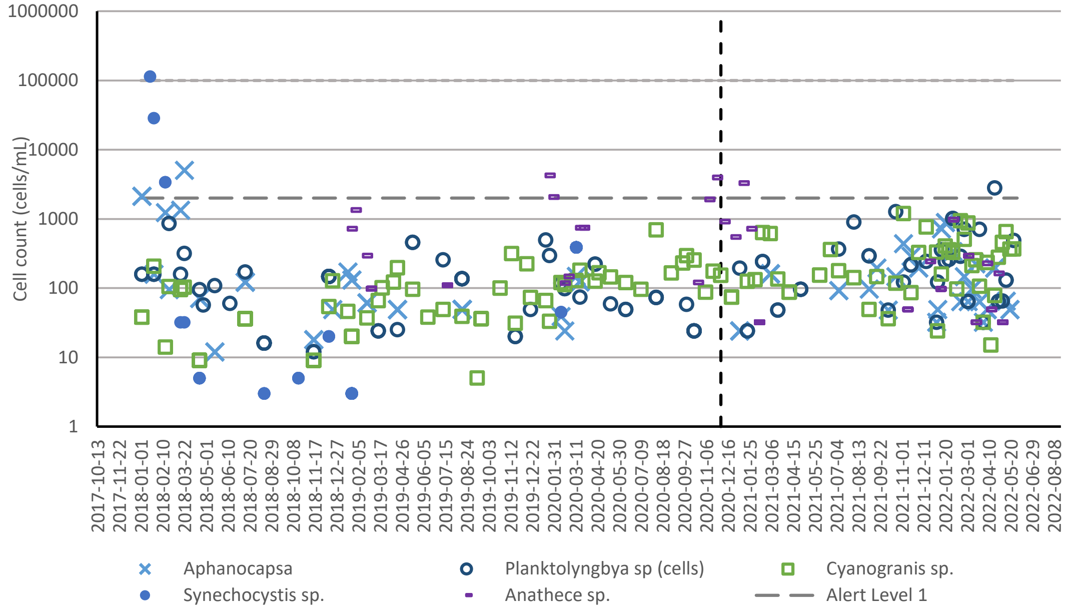

Figure S5. Reservoir C cyanobacteria cell count (dominant species) timeseries using the reservoir water dataset. Top four dominant species determined based upon cell count averages across entire dataset.

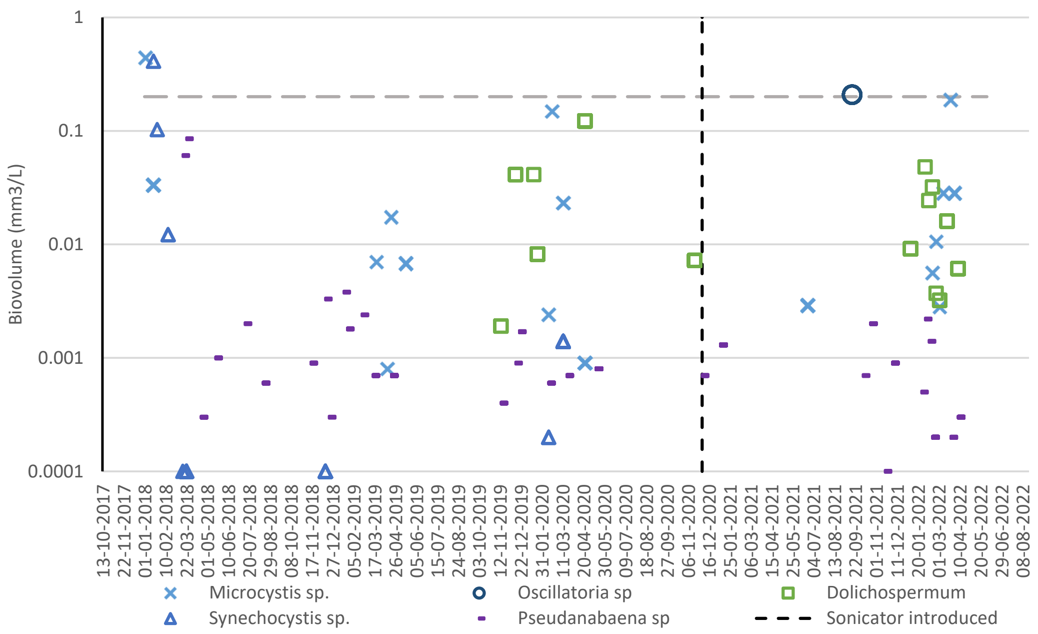

Figure S6. Reservoir C cyanobacteria biovolume (dominant species) timeseries using the reservoir water dataset. Top four dominant species determined based upon biovolume averages across entire dataset.

Table S1. Log-changes between pre- and post-sonication populations, and p-values from each t-test.

Location	Data source	Season	n (pre)	n (post)		Cyano (BV)	Cyano (CC)	Green Algae	Flagellates	Diatoms
Reservoir C	DWTP intake	Bloom	83	47	Change	-0.421	-0.433	0.667	0.847	0.176
					Significance	0.121	0.019	0.001	0.000	0.448
		Non-bloom	11	6	Change	0.761	0.640	2.385	0.634	3.773
					Significance	0.33	0.272	0.008	0.009	0.000
	Reservoir water	Bloom	42	33	Change	-0.178	-0.108	0.921	0.576	0.314
					Significance	0.656	0.747	0.005	0.032	0.274
		Non-bloom	18	11	Change	1.823	1.543	1.643	0.409	2.749
					Significance	0.021	0.009	0.004	0.124	0.000
Reservoir B	Reservoir water	Bloom	16	9	Change	-1.467	-0.858	0.089	0.651	1.787
					Significance	0.728	0.842	0.195	0.926	0.485
		Non-bloom	4	8	Change	0.792	0.303	0.839	0.063	-0.325
					Significance	0.728	0.842	0.195	0.926	0.485
Reservoir A	Reservoir water	Bloom	14	8	Change	0.042	-0.407	-0.024	-0.353	-0.338
					Significance	0.948	0.552	0.968	0.346	0.575
		Non-bloom	4	8	Change	-0.946	0.513	0.804	-0.665	0.361
					Significance	0.416	0.485	0.322	0.021	0.459
Reservoir D	Reservoir water	Bloom	8	16	Change	0.498	0.457	-0.550	-0.773	0.089
					Significance	0.492	0.420	0.240	0.335	0.887
		Non-bloom	3	10	Change	-2.899	-1.765	-0.450	0.365	-0.718
					Significance	0.017	0.029	0.530	0.284	0.017
